# Fetal Noncompaction Cardiomyopathy and Histologic Diagnosis of Spongy Myocardium: Case Report and Review of the Literature

**DOI:** 10.1055/s-0038-1673677

**Published:** 2018-10-11

**Authors:** Luigi Nappi, Lorenzo Vasciaveo, Felice Sorrentino, Gennaro Scutiero, Piergiorgio Iannone, Pantaleo Greco

**Affiliations:** 1 Department of Medical and Surgical Sciences, Institute of Obstetrics and Gynecology, Università di Foggia, Foggia, Italy; 2 Section of Obstetrics and Gynecology, Department of Morphology, Surgery and Experimental Medicine, Università degli Studi di Ferrara, Ferrara, Italy

**Keywords:** noncompaction cardiomyopathy, spongy myocardium, left ventricular noncompaction cardiomyopathy

## Abstract

Noncompaction cardiomyopathy (NCCM) and left ventricular noncompaction (LVNC), in their isolated form, are rare cardiomyopathies. They are characterized by a thickened myocardium due to the presence of deep trabeculae recesses, and to thick trabeculae. This condition is associated with a variable clinical phenotype including heart failure, thromboembolism, and sudden death. We report a case of LVNC at 26 weeks and 4 days of gestation revised on the basis of what is currently reported in the literature. A review of the literature was performed to better describe this rare condition. Left ventricular noncompaction is a rare fetal condition and it should be suspected in case of cardiomyopathy.

## Introduction

Noncompaction cardiomyopathy (NCCM) is a rare disorder that is considered to be an unclassified cardiomyopathy according to the European Society of Cardiology (ESC) Working Group on Myocardial and Pericardial Diseases and the World Health Organization (WHO), or a genetic cardiomyopathy according to the American Heart Association (AHA).^1^ ^2^ ^3^

Noncompaction cardiomyopathy mainly involves the left ventricle (left ventricular noncompaction [LVNC]), and, less frequently, both ventricles (NCCM); when it involves the right ventricle, which is rare, it shows a worse prognosis. There is a high rate of intrauterine and early neonatal deaths and the prognosis is particularly serious for hydropic fetuses. Noncompaction cardiomyopathy can be diagnosed in utero, and the main diagnostic tool is echocardiography.^4^

Left ventricular noncompaction has been diagnosed and described during the fetal life in very few case reports: only one study shows the echocardiography myocardial evaluation during the antenatal diagnosis, and recently, a small case series of nine cases was published.^5^ We report a case of antenatal diagnosis of LVNC with no other cardiac or extracardiac anomalies at 26 weeks of gestation.

## Case Description

**Fig. 1:**
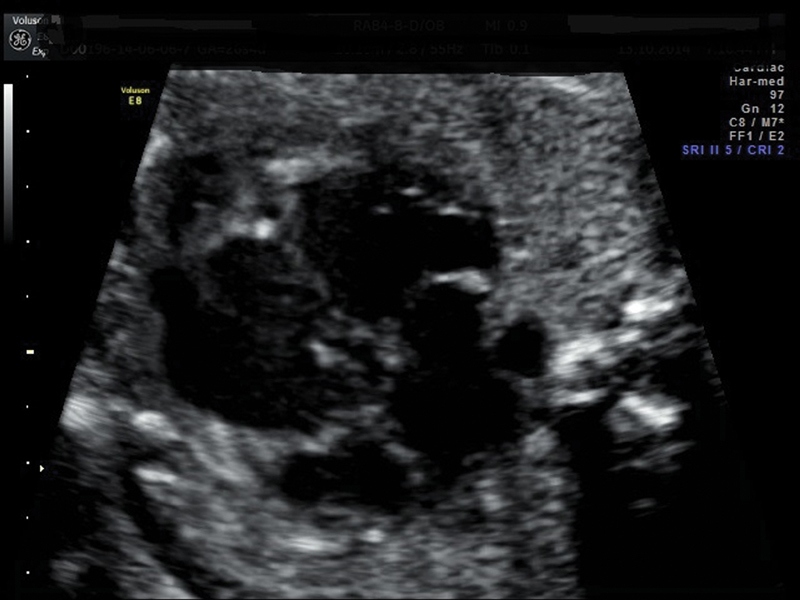
Both ventricles appear dilated with enlarged ventricular cavity biometry. Cardiac walls appear thickened and irregular on the endocardial side, especially in the apical area. First magnification.

**Fig. 2:**
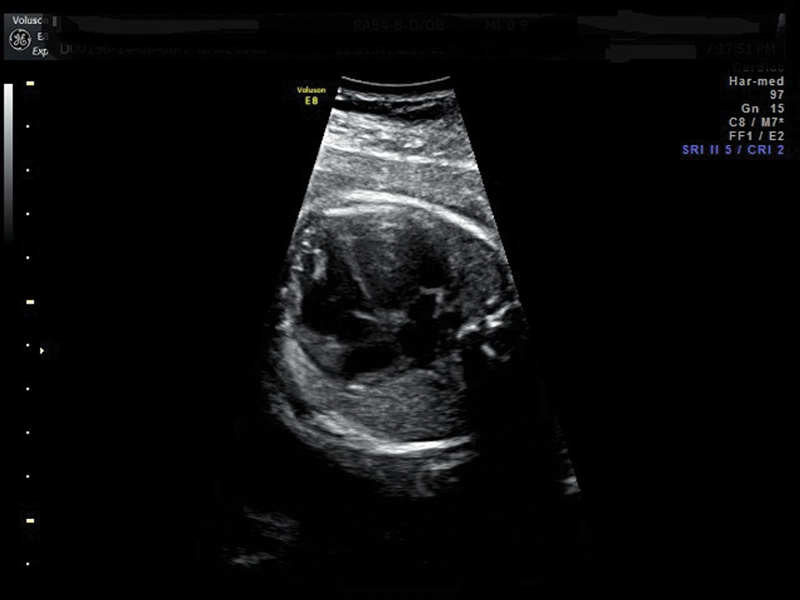
Both ventricles appear dilated with enlarged ventricular cavity biometry. Cardiac walls appear thickened and irregular on the endocardial side, especially in the apical area. Second magnification.

A 23-year-old patient, para 1, with a silent medical history, underwent a combined test during the first trimester, resulting in low risk for trisomy 21. The second trimester ultrasound, at 21 weeks, showed no cardiac anomalies in the 4 chambers, in the long axes, and in the 3-vessel views. At 26 weeks and 4 days, an ultrasound scan showed normal fetal biometry with the presence of cardiomyopathy, characterized by the heart circumference occupying more than ⅓ of the thoracic circumference: cardio-thoracic ratio > 95^th^ centile. Both ventricles appeared dilated with ventricular cavity biometry > 95^th^ percentile, with thickened and irregular walls on the endocardial side, especially in the apical area (Figs. 1 and 2). A color Doppler exam showed the presence of trabecular accentuation, protruding in the ventricular cavities due to the presence of color signal in the myocardial thickness. The absence of other structural cardiac anomalies was confirmed, and the Doppler velocimetry of the heart appeared normal, with no tricuspid valve regurgitation. Due to the new diagnosis, the second trimester ultrasound images were revised, and a quantitative evaluation of the fetal cardiac biometry was made according to the Shapiro Tables (1998). This evaluation, though limited, allowed to verify that the cardiac structures showed a normal biometry with non-harmonic values between them. The results of an invasive antenatal diagnosis and of infection investigations were negative. An echocardiography was performed on both parents with negative results. The medical history of the family was unremarkable, with no signs of cardiomyopathy.

The patient was monitored until the onset of tricuspid valve regurgitation, which led to a cesarean delivery at 31 weeks of gestation. The newborn died 3 days later. The autopsy confirmed the absence of cardiac and extracardiac anomalies. The diagnosis was idiopathic NCCM or “spongy myocardium.”

## Discussion

Noncompaction cardiomyopathy refers to an uncommon structural abnormality of the heart's ventricular myocardium characterized by an abnormally thick layer of left ventricular trabeculations, as well as hypoplasia of the papillary muscles. It is a rare pathology with great etiology and clinical management difficulties. It is associated with a variable clinical phenotype including heart failure, thromboembolism, and sudden death.^6^ Noncompaction cardiomyopathy is a relatively recent addition to the diagnostic catalogue. Currently, it is controversial whether NCCM is a distinct cardiomyopathy or a morphological characteristic shared by different heart diseases.

Thus, this rare disorder is considered to be an unclassified cardiomyopathy according to the ESC Working Group on Myocardial and Pericardial Diseases and the WHO (it is not clear whether it is a separate cardiomyopathy or merely a morphological trait shared by many phenotypically distinct cardiomyopathies), or a genetic cardiomyopathy according to the AHA^1^ ^2^ ^3^ Echocardiography is the main method for detecting NCCM.^4^

Left ventricular non-compaction comprises the majority of the cases, but NCCM can also be seen in the right ventricle or in both ventricles.^5^

Left ventricular noncompaction affects the left ventricular myocardial structure by the compaction process interruption between the 5^th^ and the 8^th^ gestational week.^7^ At this time, the myocardium is widely formed by trabeculae because the coronary system is not yet developed and the wide intertrabecular spaces allow the blood flow to spread to the myocardial developing tissues. Between the 5^th^ and the 8^th^ week, the trabeculae regress following the myocardial fiber compaction. This process occurs from the basis to the cardiac apex, while the coronary system develops, supporting the growing myocardial tissue. According to the pathogenetic theory, myocardial morphogenesis interruption occurs, preventing the trabecular regression and muscle tissue compaction, although the coronary system is normally developed. This leads to a gradual myocardial fiber compaction failure, determining an excessive trabecular formation with deep ventricular wall recesses. These remarkable trabeculations, especially in the ventricular vertex, are responsible for the anomalous aspect of the ventricular cavity.^7^ ^8^ ^9^

Histopathology has shown a continuity between the endothelium of the intertrabecular recesses and that of the endocardium, distinguishing LVNC from persistent sinusoids. Other findings have included loosely organized myocytes and endocardial and subendocardial replacement fibrosis, suggestive of ischemic necrosis. Left ventricular dilatation and ischemia are frequently present, and thrombus formation in the recesses may occur, which may be associated with possible thromboembolic events. Since there is no gold standard for the diagnosis, the sensitivity and specificity of the morphologic criteria are uncertain. The Jenni et al^10^ criteria are the most widely accepted. These criteria were developed based upon the echocardiographic appearance with pathologic confirmation in seven patients with LVNC; the criteria were validated in a second population.^10^

In conclusion, the diagnosis of LVNC is based on these morphologic and echocardiographic criteria:

- Thickened myocardium with a two-layered structure consisting of a thin compacted epicardial layer/band (C) and a much thicker, non-compacted endocardial layer (N) or trabecular meshwork with deep endomyocardial spaces; N/C ratio of 2:0 at end-systole in the parasternal short-axis view
- Hypoplasia of the papillary muscles;
- Noncompaction anomalies involving the lateral, inferior and apical myocardial segments;
- Color Doppler evidence of flow within the deep intertrabecular recesses;^8^ ^10^
- Trabeculae and intertrabecular recesses are covered by the endocardium, filled with blood with no communication with the coronary circulation.^8^

The clinical relevance of NCCM associated to other cardiac anomalies is still not well defined. One hypothesis, based on recent research, says that families affected by LVNC show mutations of the sarcomeric protein coding genes and myosin heavy chain in continuum with hypertrophic, restrictive cardiomyopathy and, more rarely, with dilated cardiomyopathy.^11^ ^12^

Left ventricular noncompaction is almost invariably associated with other congenital cardiac malformations, including atrioventricular canal defects, double-outlet right ventricle, valvular atresia, ventricular septal defect, and transposition of the great arteries.^13^

The clinical presentation varies from no symptoms to heart failure, embolism, arrhythmia, mitral insufficiency, conduction disorders and sudden death.^14^ Patients (fetus, child or adult) who show heart failure symptoms tend to have a diminished ventricular function and a poor prognosis; those identified with family screening or with echocardiography usually have a less serious disease, no symptoms and a good prognosis in adult life. Noncompaction cardiomyopathy cases diagnosed in utero are very few, with almost no data about long-term follow-ups.^8^

A retrospective study of autopsied fetuses and neonates with NCCM showed that heart failure, including heart block, is a common cause of death. NCCM is often associated with various cardiovascular malformations, but even in isolation it can be the basis for severe cardiac failure, and biventricular endocardial fibroelastosis in NCCM suggests a global pathologic process.^6^

The differential diagnosis of LVNC is based on: false tendons, remarkable trabeculae as normal variants, apical hypertrophic cardiomyopathy, dilated cardiomyopathy with no spongy myocardium, Fabry disease, right arrhythmogenic ventricular dysplasia, and endocardial fibroelastosis. Patients with LVNC should be submitted to screening for: congenital cardiac defects, genetic anomalies, and neuromuscular and metabolic diseases.^8^ ^12^

## Final Considerations

Ultrasound technical improvements and the progress in the training of ultrasound operators have augmented the cardiac structure detection during fetal echocardiography. For this reason, NCCM should be considered for a differential diagnosis in cases of dilated cardiomyopathy.^15^ The actual debate of a defined separation between LVNC and other overlapping forms of cardiomyopathies is still under discussion, requiring further studies.^9^ We hypothesize that LVNC belongs to the group of cardiomyopathies in which every entity shows aspects that can overlap with the others and may share a common genetic basis. This possibility gives new research perspectives on the etiopathogenetic mechanisms of genetic myocardial diseases.

## References

[1] Maron B J Towbin J A Thiene G Contemporary definitions and classification of the cardiomyopathies: an American Heart Association Scientific Statement from the Council on Clinical Cardiology, Heart Failure and Transplantation Committee; Quality of Care and Outcomes Research and Functional Genomics and Translational Biology Interdisciplinary Working Groups; and Council on Epidemiology and Prevention Circulation 2006 113 14 1807 1816 Doi: 10.1161/CIRCULATIONAHA.106.174287 16567565 10.1161/CIRCULATIONAHA.106.174287

[2] Elliott P Andersson B Arbustini E Classification of the cardiomyopathies: a position statement from the European Society Of Cardiology Working Group on Myocardial and Pericardial Diseases Eur Heart J 2008 29 02 270 276 Doi: 10.1093/eurheartj/ehm342 17916581 10.1093/eurheartj/ehm342

[3] Fuster V The 3 pathways of translational medicine: an evolution to a call-and-response method J Am Coll Cardiol 2014 64 02 223 225 Doi: 10.1016/j.jacc.2014.06.002 25011725 10.1016/j.jacc.2014.06.002

[4] van Velzen C L Clur S A Rijlaarsdam M E Prenatal diagnosis of congenital heart defects: accuracy and discrepancies in a multicenter cohort Ultrasound Obstet Gynecol 2016 47 05 616 622 Doi: 10.1002/uog.15742 26350159 10.1002/uog.15742

[5] Tian L Zhou Q Zhou J Zeng S Cao D Zhang M Ventricular non-compaction cardiomyopathy: prenatal diagnosis and pathology Prenat Diagn 2015 35 03 221 227 Doi: 10.1002/pd.4523 25346355 10.1002/pd.4523

[6] Ursell P C Noncompaction in the fetus and neonate: an autopsy study Am J Med Genet C Semin Med Genet 2013 163C 03 169 177 Doi: 10.1002/ajmg.c.31367 23720434 10.1002/ajmg.c.31367

[7] Sedmera D Thomas P S Trabeculation in the embryonic heart BioEssays 1996 18 07 607 Doi: 10.1002/bies.950180714 8757939 10.1002/bies.950180714

[8] Tsapakis E G Eleftheriades M Daskalakis G Chrelias C Hassiakos D Prenatal diagnosis of fetal left ventricular non-compaction cardiomyopathy Ultrasound Obstet Gynecol 2012 39 05 592 594 Doi: 10.1002/uog.9086 21728209 10.1002/uog.9086

[9] Seidman J G Seidman C The genetic basis for cardiomyopathy: from mutation identification to mechanistic paradigms Cell 2001 104 04 557 567 Doi: 10.1016/S0092-8674(01)00242-2 11239412 10.1016/s0092-8674(01)00242-2

[10] Jenni R Oechslin E Schneider J Attenhofer Jost C Kaufmann P A Echocardiographic and pathoanatomical characteristics of isolated left ventricular non-compaction: a step towards classification as a distinct cardiomyopathy Heart 2001 86 06 666 671 Doi: 10.1136/heart.86.6.666 11711464 10.1136/heart.86.6.666 PMC1730012

[11] Hoedemaekers Y M Cohen-Overbeek T E Frohn-Mulder I M Dooijes D Majoor-Krakauer D F Prenatal ultrasound diagnosis of MYH7 non-compaction cardiomyopathy Ultrasound Obstet Gynecol 2013 41 03 336 339 Doi: 10.1002/uog.12279 22859017 10.1002/uog.12279

[12] van der Linde I HM Hiemstra Y L Bökenkamp R A Dutch MYH7 founder mutation, p.(Asn1918Lys), is associated with early onset cardiomyopathy and congenital heart defects Neth Heart J 2017 25 12 675 681 Doi: 10.1007/s12471-017-1037-5 28864942 10.1007/s12471-017-1037-5 PMC5691818

[13] Arunamata A Punn R Cuneo B Bharati S Silverman N H Echocardiographic diagnosis and prognosis of fetal left ventricular noncompaction J Am Soc Echocardiogr 2012 25 01 112 120 Doi: 10.1016/j.echo.2011.09.019 22014428 10.1016/j.echo.2011.09.019

[14] Karatza A A Holder S E Gardiner H M Isolated non-compaction of the ventricular myocardium: prenatal diagnosis and natural history Ultrasound Obstet Gynecol 2003 21 01 75 80 Doi: 10.1002/uog.10 12528168 10.1002/uog.10

[15] Yinon Y Yagel S Hegesh J Fetal cardiomyopathy--in utero evaluation and clinical significance Prenat Diagn 2007 27 01 23 28 Doi: 10.1002/pd.1612 17154225 10.1002/pd.1612

